# Electrosprayed Chitosan–Copper Complex Microspheres with Uniform Size

**DOI:** 10.3390/ma14195630

**Published:** 2021-09-28

**Authors:** Andrea Lončarević, Marica Ivanković, Anamarija Rogina

**Affiliations:** Faculty of Chemical Engineering and Technology, University of Zagreb, Marulićev trg 19, 10000 Zagreb, Croatia; mivank@fkit.hr (M.I.); arogina@fkit.hr (A.R.)

**Keywords:** chitosan, copper, complex, microspheres, electrospray

## Abstract

Chitosan-based nano- and microspheres have shown great potential in a broad range of applications, including drug delivery, bone tissue engineering, wastewater treatments, etc. The preparation of uniformly sized spheres with controlled morphology and microstructure is still a challenge. This work investigates the influence of cupric ions (Cu^2+^) on the size, shape, morphology and stability of electrosprayed chitosan–copper (CHT–Cu^2+^) complex microspheres, using chitosans with different degrees of deacetylation. The dynamic viscosity of CHT–Cu^2+^ solutions was measured by Höppler viscometer, while attenuated total reflectance Fourier transform infrared spectroscopy (ATR-FTIR) was used for the identification of dried microspheres. The size, shape and morphology of microspheres were analyzed by light microscope and scanning electron microscopy (SEM), while stability of dried microspheres was evaluated in different buffer solutions. The volume ratio of wet and dry microspheres was assessed based on the estimated diameter of microspheres. The higher concentration of Cu^2+^ ions resulted in a decrease in viscosity of CHT–Cu^2+^ solutions and volume ratio of prepared microspheres. Changes in the intensities and wave numbers of absorption bands of amino and hydroxyl groups, amide I and amide II suggested that the nitrogen and oxygen atoms in chitosan are coordinating the cupric ions. Micrographs obtained by light microscope and SEM showed that all prepared samples are spherical. The increase of cupric ions concentration changed the topography of microspheres and decreased their size. These results indicated the successful electrospraying of CHT–Cu^2+^ microspheres with uniform size and good stability in aqueous medium.

## 1. Introduction

Chitosan-based nano- and microspheres have versatile applications such as smart drug and gene delivery carriers with controlled release in biomedicine [1,2,3,4], in bone tissue engineering [5,6], as adsorbents in the removal of metals, radionuclides and dyes in wastewater treatments [7,8,9,10,11], in food and agriculture industry [12,13], etc. The traditional methods of preparing chitosan-based microspheres are single or double emulsion techniques, coacervation/precipitation, solvent evaporation and ionic gelation [2,3,14,15,16].

Chitosan is a cationic polysaccharide, biocompatible, biodegradable, bioabsorbable, non-toxic biopolymer with antibacterial, antifungal and hemostatic properties [2,8,9,15,17,18]. It can be obtained by the alkali *N*-deacetylation of chitin, the second most abundant polysaccharide in the world, which can be found in crustaceans, insects and fungi. The degree of deacetylation (*DD*) and molecular weight of chitosan are important parameters in the determination of its physicochemical properties [2,6,15]. The amino groups (–NH_2_) in the macromolecules’ chains are protonated (–NH_3_^+^) in an acidic medium, resulting in a soluble polycation that has a high charge density. Due to the presence of the positive charge, chitosan can form complexes with other (bio)polymers that have a total negative charge (e.g., polyanionic nucleic acids) [4,18,19], as well as anionic compounds like metal anions or anionic dyes [9]. Chitosan macromolecules, containing a large number of amino and hydroxyl groups, could form complexes with transition metal ions through various mechanisms such as chelation, electrostatic attraction or ion exchange, depending on the metal ion and the pH of the solution. The main complex sites are nitrogen and oxygen atoms owing to electron pairs that could be combined with metal ions [9,13,20]. One of the transition metals that possess strong chelation with amine is copper. There are few possible models of the interaction between chitosan and copper (II) ions. The first model suggests that a cupric ion is bound with four amino groups in a square-planar geometry [21]. The second model proposes that chitosan’s binding sites are two amino groups and two hydroxyl groups [22]. Furthermore, Guibal [23] also proposed two other chitosan–copper (II) ion complex models. The first one is the pendant model, and the second one is the bridge model. In the pendant model, the Cu^2+^ ion is connected to one amino group, while in the bridge model the Cu^2+^ ion interacts with two or more amino groups from the same or different polymer chain by the intra- or intermolecular complexation.

Electrospraying or electrohydrodynamic atomization is a modern, one-step process for obtaining nano- and microspheres [3]—hollow or solid spherical structures in range size from tens of nanometers to a few millimeters [24]. The basic principle of the electrospraying process lies in applying high voltage on a droplet that undergoes breaking-up into smaller droplets when electrostatic force inside the droplet (Coulomb force) overcame the cohesive force of the droplet (surface tension). This begins at the Taylor cone, which is the characteristic phenomenon of a charged droplet. The resulting smaller droplets are well dispersed with narrow size distribution and do not coalesce during their travel toward the collector due to Coulomb repulsion of the charges [14,25]. The advantages of the electrospraying method with respect to the conventional methods are the possibility of using high molecular weight polymers, production of nanoparticles, utilization of fewer organic solvents and non-use of surfactants or crosslinkers. The process is one-step, simple, low cost, reproducible, conducted at ambient conditions and generates less residue. Furthermore, prepared microspheres are homogeneous in size distribution and morphology [2,3,12,14].

Although chitosan serves as a suitable polymer for producing nano- and microsized particles, their stability in aqueous environment is quite poor. Due to its hydrogel nature, chitosan swells in aqueous solution with high swelling capacity in diluted acidic solution which usually ends in dissolution of chitosan-based materials. To solve this problem, many researchers [26,27,28] have been focused on modifying the chemical structure of chitosan by using different types of crosslinkers (e.g., glutaraldehyde and genipin) which could improve chitosan stability in acidic environments and reduce the capacity of water absorption. However, such procedures involve toxic chemicals which could remain within chitosan after crosslinking reaction. Hence, this work highlights the alternative for producing stable chitosan microspheres by using simple complexation reactions through transition metal ions. The great affinity of copper (II) ions towards amino and hydroxyl groups could be responsible for the formation of chitosan–Cu^2+^ complexes that preserve the hydrogel structure of chitosan in slightly acidic environment. The influence of different concentrations of cupric ions (Cu^2+^) on the size, shape and morphology of electrosprayed chitosan–copper complex microspheres has been investigated. The potential of chitosan–Cu^2+^ physical crosslinking has been demonstrated through stable microspheres with uniform size produced at higher concentration of copper (II) ions.

## 2. Materials and Methods

### 2.1. Materials

In this work, two types of chitosan were used, both with medium molecular weight declared by the manufacturer, and with different degrees of deacetylation (*DD*). Chitosan with *DD* = 85% (CHT85) was purchased from Sigma-Aldrich (Saint Louis, MO, USA), while chitosan with *DD* = 95% (CHT95) was purchased from Acros Organics (Geel, Belgium).

Copper acetate monohydrate (BDH Prolabo, Leuven, Belgium) was used as a precursor of copper (II) ions (Cu^2+^) for the complexes’ preparation. Other chemicals for materials preparation were 99.8% acetic acid (HAc; Lach–Ner, Neratovice, Czech Republic), sodium hydroxide (NaOH; Honeywell, Seelze, Germany), 96% ethanol (EtOH; Kefo, Ljubljana, Slovenia) and acetone (T.T.T. doo, Sveta Nedjelja, Croatia). Sodium acetate trihydrate (NaAc) purchased from Sigma-Aldrich (Saint Louis, MO, USA) and potassium dihydrogen phosphate (KH_2_PO_4_) purchased from Gram mol (Zagreb, Croatia) were used to prepare the buffer solutions. All chemicals were of analytic grade.

### 2.2. Preparation of Solutions for Electrospraying Process

Firstly, 1.2 wt.% chitosan solution was prepared by dissolving polymer powder in 0.5% solution of acetic acid for 24 h at ambient conditions. The obtained chitosan solution was then filtered to remove possible impurities and insoluble parts. This process was the same for both chitosans (CHT85 and CHT95).

Next, solutions of copper (II) ions (Cu^2+^) at different concentrations were prepared. As described in detail in our previous work [29], the amount of Cu^2+^ ions was added with respect to the ratio of Cu^2+^ ions and amino groups in chitosan as follows: *n*(Cu^2+^):*n*(–NH_2_) = 0.009:1, 0.018:1 and 0.09:1.

The chitosan–copper (II) ions complex solutions (CHT–Cu^2+^) were prepared by mixing the appropriate volume of chitosan solution with Cu^2+^ solution while keeping the volume ratio v(chitosan)/v(Cu^2+^) to 7.33 and stirring for 2 h. Prepared CHT–Cu^2+^ solutions were denoted as CHT–Cu0.5, CHT–Cu1 and CHT–Cu5, respectively, for both chitosans (*DD* of 85 and 95%).

Furthermore, 5 wt.% sodium hydroxide solution (NaOH) was prepared by dissolving the proper amount of NaOH in distilled water and was used as a gelation medium during electrospraying process.

All solutions were freshly prepared for each electrospraying process.

### 2.3. Preparation of Chitosan–Metal Complex Microspheres

**Figure 1:**
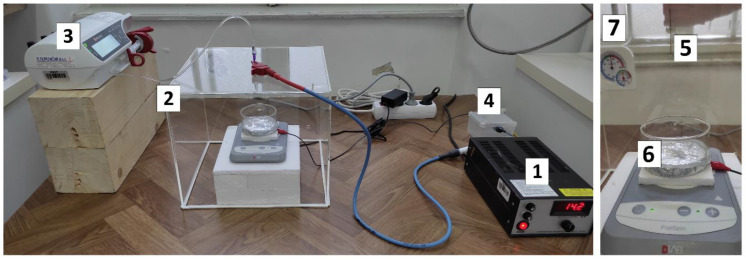
Electrospraying apparatus: (1) high voltage generator, (2) poly(methyl- methacrylate) chamber, (3) syringe pump with different needle gauges for feeding the test solution and (4) grounding. In the chamber were: (5) metal needle with obtained Taylor cone-jet, (6) collector (crystallizing dish with flat bottom, with 50 mL of 5 wt.% NaOH solution and magnetic stirring bar, isolated with aluminum foil) and (7) temperature and relative humidity sensor.

The electrospraying process was conducted using the apparatus shown in Figure 1 with the constant process parameters given in Table 1.

The electrospraying process was conducted as follows: the syringe (Becton Dickinson, Le Pont-de-Claix, France) was filled with prepared chitosan–Cu^2+^ complex solution (10 mL) and processing parameters presented in Table 1 were set. The needle was positively charged and the collector was grounded. Crystallizing dish with 50 mL of 5 wt.% NaOH solution (gelation medium) isolated with aluminum foil was used as the collector. The distance between the surface of the NaOH solution and the blunt tip needle was set at 10 cm. The required voltage for the electrospraying process was determined according to the formation of stable Taylor cone-jet mode. For CHT85-based complex solutions, stable jet was observed at the voltage of 23 kV, and for CHT95-based solutions at 14 kV. The flow rate of solutions was 5 mL h^−1^ and the process was conducted for 1 h (without interruption) with gentle stirring of gelation medium. After that, gelation medium with formed hydrogel microspheres was stored in the falcon tubes for the next 24 h. Microspheres were then washed with distilled water until pH neutral and left in the water for 24 h. Thereafter, microspheres were dehydrated with 96% EtOH for 24 h and dried with acetone (acetone exchanged three times) and left at the ambient conditions for solvent evaporation.

### 2.4. Preparation of Buffer Solutions for Stability Study

The stability of chitosan-based microspheres was evaluated in different buffers: in acetic acid–sodium acetate (HAc–NaAc) buffer solution as an acidic medium, and potassium dihydrogen phosphate–sodium hydroxide (KH_2_PO_4_–NaOH) buffer solution as mild acidic and neutral medium.

HAc–NaAc buffer solution with pH 5.0 was prepared by dissolving a proper amount of NaAc in 40 mL of distilled water and adding the proper amount of HAc while stirring. pH value of prepared solution was measured and adjusted to 5.0 by adding 15 drops of 5 wt.% NaOH solution. Finally, distilled water was added until the buffer solution’s volume was 50 mL.

KH_2_PO_4_–NaOH buffer solutions (pH 6.0 and 7.0) were prepared by dissolving a proper amount KH_2_PO_4_ in 80 mL of distilled water followed by dissolving a proper amount of NaOH while stirring. Solution with pH 6 was adjusted by the addition of 5 drops of 5 wt.% NaOH solution. Finally, distilled water was added until the buffer solution’s volume was 100 mL, for both prepared buffer solutions.

All prepared buffer solutions were left for 30 min at ambient conditions before use.

### 2.5. Characterization of Chitosan–Cu^2+^ Solutions

The density (*ρ*, g cm^−3^) of prepared solutions was measured by the hydrometer (Greiner Glasinstrumente, Lemgo, Germany) at 20 °C.

The dynamic viscosity (*η*, mPa s) of prepared solutions was measured by falling-ball Höppler viscometer (VEB MLW Prüfgeräte-Werk Medingen, Sitz Freital, Freital, Germany) at 20 °C and calculated using Equation (1):

*η* = *t* × (*ρ*_ball_ − *ρ*_solution_) × *K*, (1)

where *t* (s) is the travel time of the ball, *ρ*_ball_ (g cm^–3^) is the density of the ball, *ρ*_solution_ (g cm^−3^) is the density of the test solution and *K* (mPa cm^3^ g^–1^) is a constant of the ball. The measurements were performed in triplicates and the results are shown as mean value with standard deviation.

### 2.6. Characterization of Prepared Microspheres

Attenuated total reflectance Fourier transform infrared spectroscopy (ATR-FTIR; Bruker Vertex 70, Bruker Optics, Ettlingen, Germany) was used for the identification of dried microspheres at 20 °C. The wave number range was from 4000 to 800 cm^−1^, with 32 scans and a spectral resolution of 2 cm^−1^. The dried microspheres were powdered before analyzing if needed.

The wet and dried microspheres were analyzed with a BA200 binocular microscope (light microscope; Motic Instruments, Barcelona, Spain). Pictures were taken using the Motic Images Plus 2.0 program and processed by ImageJ 1.53e software which was used for the estimation of microspheres’ diameters. The diameter was calculated from the area of at least 20 randomly chosen microspheres, assuming total sphericity of microspheres.

The morphology of dried microspheres was investigated by scanning electron microscopy (SEM; Tescan Vega III Easyprobe, Brno-Kohoutovice, Czech Republic) with electron beam energy of 10 keV. Before imaging, samples were exposed to the plasma of gold and palladium for 75 s.

Based on the estimated diameter, the volume ratio of wet microspheres in distilled water, *V*_wet_ (μm^3^), and dried microspheres obtained after the process of dehydration and drying, *V*_dry_ (μm^3^), was assessed (as estimated water content of microspheres).

Stability studies were conducted on selected chitosan–Cu^2+^ microspheres immersed in different buffer solutions in closed chamber at temperature of 26 ± 1 °C and relative humidity of 71 ± 2% for 24 h. Microspheres were put on the microscopic slide and 2–3 mL of buffer solution was added. Pictures were taken by light microscope immediately after the microspheres’ exposure to the buffer solution and after 24 h of immersion.

## 3. Results and Discussion

### 3.1. ATR-FTIR Analysis of Microspheres

**Figure 2:**
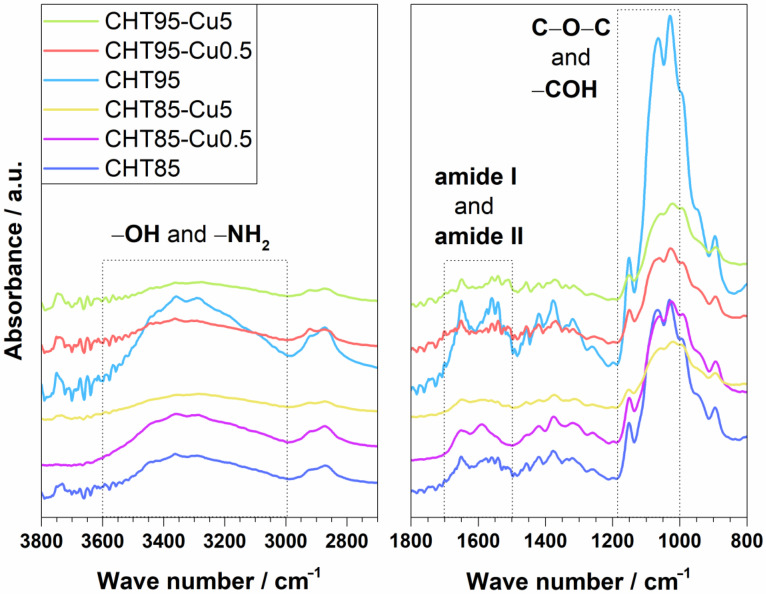
ATR–FTIR spectra of pure chitosans and prepared CHT–Cu^2+^ microspheres.

ATR-FTIR spectroscopy was used for the identification of obtained dried CHT–Cu^2+^ microspheres. Figure 2 shows spectra of pure chitosans and complexes with the lowest (Cu0.5) and highest concentration (Cu5) of cupric ions. Absorption bands of functional groups of CHT85, CHT95 and their CHT–Cu^2+^ complexes are presented in Table 2.

As can be seen from Figure 2 and data in Table 2, the absorption bands characteristic for chitosan were found at 3362 and 3290 cm^−1^ for CHT85 and at 3359 and 3292 cm^−1^ for CHT95, which correspond to the stretching vibration of the hydroxyl group (–OH) and the extension vibration of the amino group (–NH_2_) and their interactions through hydrogen bonds [7,20]. Two bands observed in the range of 2950–2850 cm^−1^ are associated with the symmetric and asymmetric stretching of C–H in –CH– and –CH_2_ [8,13]. Absorption bands at ~1650, 1541 cm^−1^ and in the range of 1350–1200 cm^−1^ are assigned to amide I (stretching vibrations of C=O with C–N stretching and N–H bending in –CONH_2_), amide II (C–N stretching vibrations in combination with N–H bending) and amide III (N–H bending with C–N stretching, and C–H and N–H deformation vibrations), respectively [30]. The asymmetric stretching of C–O–C appears at 1150 cm^−1^, while in the range of 1070–1010 cm^−1^ –CO stretching vibrations in –COH (on C3 atom, secondary –OH) are present [7,8,29].

The changes in intensities and wave numbers of bands of amino and hydroxyl groups, amide I and amide II are considered as indicators of chelation of metal ions with –NH_2_ and –OH groups [29,31]. The intensity of the broad band at 3700–3000 cm^−1^ decreased and the band was shifted to lower wave numbers when copper (II) ions were added into chitosan (CHT85 and CHT95). This could indicate that amino and hydroxyl groups are involved in the chelation process since cupric ions have affinity towards those functional groups. Similar results were obtained by Ren et al. [7] where chromium (VI) ions were adsorbed onto porous chitosan microspheres. Furthermore, the changes of intensity of absorption bands characteristic for amide I, amide II and C–O–C vibrations were observed, while absorption band characteristic for –COH was shifted to lower wave numbers, as presented in Table 2. In addition, absorption band characteristic for amide III was difficult to observe with the addition of cupric ions in CHT85–Cu complexes, which could be due to bands overlapping. In CHT95–Cu complexes amide III band was slightly shifted to higher values.

The observed changes in the intensities and wave numbers of absorption bands could suggest that the nitrogen and oxygen atoms in chitosan functional groups are coordinating the cupric ions. Furthermore, all spectra were recorded using dried complex microspheres which indicates stable CHT–copper (II) complexes after several steps of dehydration and drying [13]. The presented results are in agreement with our previous work [29], where porous CHT–metal ion scaffolds were prepared.

Although Zhang et al. [20] observed the appearance of new absorption bands after the adsorption of Cu (II) ions, the formation of new absorption bands was not detected in this work. It can be concluded that electrostatic forces between chitosan and cupric ions are the only bond formed in CHT–Cu^2+^ complex microspheres.

### 3.2. The Viscosity of Chitosan and Chitosan–Cu^2+^ Solutions

The main properties of a polymeric solution that affect the electrospraying process are its density, surface tension, viscosity, electrical conductivity, concentration and molecular weight of polymer [12]. The parameters influencing the viscosity of chitosan solution are molecular weight, *DD*, concentration of solution and used solvent. Generally, the size of microspheres increases with increasing solution viscosity [15,16]. In this work, the dominant effect on the viscosity of polymer solutions had a degree of deacetylation since concentration of chitosan solutions and solvent type were the same.

In this work, the dynamic viscosity was measured by Höppler viscometer and the results are presented in Table 3. To calculate the viscosity (Equation (1)), the density of the solution was determined just before the viscosity measurements. The density of all prepared solutions was determined to be 1.004 g cm^−3^.

The addition of cupric ions decreases the viscosity of pure polymer solutions even at the minimal concentration of copper (II) ions (Cu0.5), as seen in Table 3. It is worth noting that CHT85 had a higher viscosity than CHT95. Consequently, all CHT85-based complexes showed higher viscosity in comparison to CHT95–copper complexes, even both chitosans were declared as medium molecular weight polymers.

Decreasing viscosity was more pronounced by Cu^2+^ ions in CHT85-based complexes than for CHT95–copper (II) complexes. Compared to pure chitosan solutions, the CHT85–Cu0.5 and CHT95–Cu0.5 solutions show the viscosity decrease of 1.5 and 1.35 times, respectively. The significant drop in viscosity happened for CHT85 at the highest concentration of copper (II) ions, i.e., 2.4 times for CHT85-Cu5, while poor impact of metal ions was detected for CHT95-Cu5 (only 1.6 times lower viscosity). The more pronounced decrease in viscosity of the CHT85 solution with the addition of cupric ions could be due to the conformational changes of polymer chains. By introducing the copper (II) ions in chitosan solution, Cu^2+^ ions occupied some amino and hydroxyl groups, which are no longer involved in hydrogen bonding within the same chain or between the chains. Significant decrease in viscosity of CHT85-Cu complexes could imply that a greater number of ligands from the same or different chain was involved in the coordination of copper (II) ions. Decrease in viscosity of CHT–Cu^2+^ solutions by Cu^2+^ ions could be a result of lower electrostatic repulsions between protonated chitosan chains, assuming the successful formation of CHT85–Cu^2+^ and CHT95–Cu^2+^ complexes.

### 3.3. Estimated Size and Shape of Electrosprayed Chitosan–Cu^2+^ Microspheres

**Figure 3:**
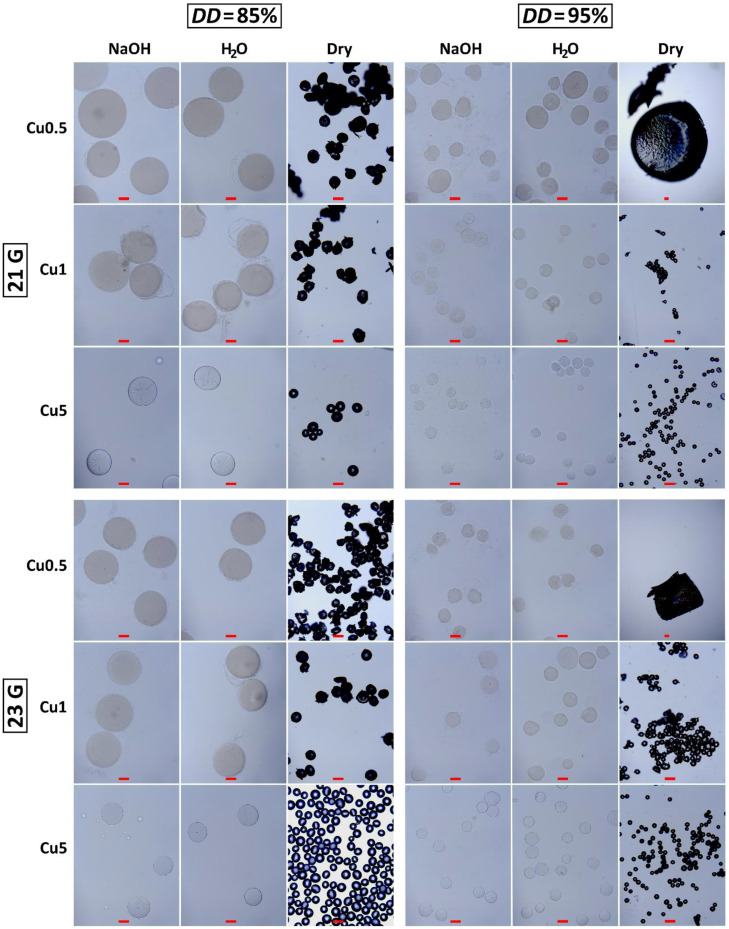
CHT85–Cu^2+^ and CHT95–Cu^2+^ microspheres in the 5 wt.% NaOH solution (gelation medium), distilled water (H_2_O) and dried microspheres obtained by the electrospraying method with two different needles gauges (21 G and 23 G). All scale bars represent 100 μm. Please note, micrographs of dry CHT95–Cu0.5 complexes were taken at lower magnification (4×).

Figure 3 shows micrographs of CHT–Cu^2+^ microspheres during each step of the electrospraying process: in alkaline medium (NaOH), distilled water (H_2_O) and microspheres obtained after drying process (dry). The concentration of polymer solution plays a critical role in electrospraying the nano- and microparticles. Chitosan microspheres are microscale hydrogels formed by physical interactions of macromolecules (electrostatic forces, hydrophobic interactions and hydrogen bond), i.e., chain entanglements. In dilute regimes, a critical overlap concentration within the polymer concentration range needs to be exceeded for the onset of chain overlapping [32]. When the solution is concentrated, chain dimensions form entanglements that are independent of the polymer concentration. The concentration of chitosan solution used in this work is above the critical concentration for the entanglement formation, as observed from successfully generated microspheres. Furthermore, copper (II) ions had an additional impact on physical interactions and crosslinking. However, it seems there is a minimal *c*(Cu^2+^) needed to preserve stable chitosan–Cu^2+^ chelate. Judging by the dried CHT95–Cu0.5 microspheres (Figure 3), lower concentration of Cu^2+^ ions was not sufficient to form microspheres that are stable after the drying process.

When it comes to collecting the droplets, there are two ways that the electrospraying process can be carried out. The first one includes a grounded plate-collector where deposited charged droplets are collected individually or as agglomerates, and the second one is when charged droplets are electrosprayed into the solution which is used as a gelation medium [12,33,34]. In this work, electrosprayed microspheres were collected in 5 wt.% NaOH solution followed by washing with distilled water, dehydration (EtOH) and drying (acetone) at ambient conditions. During the drying process, CHT85–Cu0.5 and CHT–Cu1 microsphere aggregates were formed. They were fragmented into the smaller ones or powdered by gently applying pressure with a spatula. CHT–Cu5 microspheres of both chitosans were obtained in powder form and were imaged without further processing. Furthermore, process of dehydration and drying was not adequate for CHT95 microspheres with the lowest *c*(Cu^2+^) obtained by both needles (21 G and 23 G).

CHT85- and CHT95-based microspheres were found to be spherical. The addition of cupric ions caused a slight change in the shape of microspheres. Solutions with lower *c*(Cu^2+^) (CHT–Cu0.5 and CHT–Cu1) gave slightly stretched droplets (ellipsoids), while higher metal ions concentration (CHT–Cu5) resulted in round, smooth, more spherical microspheres. It can be assumed that stronger physical crosslinking obtained by greater amount of metal ions could give homogeneous and spherically shaped microspheres. This observation is in agreement with the previous report [35]. Obtained homogeneous microsphere sizes and morphology, as well as uniform size, are necessary for many applications [14,36].

Process parameters that influence the production of microspheres are applied voltage (in kV), needle gauge, flow rate of polymer solution and distance between the needle tip and collector. Furthermore, relative humidity, pressure and temperature are important factors in the determination of the process stability [15]. In this work, applied voltage and needle gauge were chosen as variable process parameters, while others were kept constant (as presented in Table 1). Several electrospray modes can be obtained during the electrospraying process based on the applied voltage (e.g., dripping, rapid dripping, unstable cone-jet, Taylor cone-jet, multi-jet and irregular unstable mode) [16], from which the most desirable is single Taylor cone-jet mode owing to its stability, reproducibility and production of small and monodisperse microspheres [36,37]. Stable Taylor cone-jet mode was formed at the voltages of 23 and 14 kV for CHT85-based and CHT95-based complex solutions, respectively. This is in accordance with determined viscosity of solutions (Table 3): a stronger electrical field was required for CHT85-based complex solutions to form Taylor cone-jet mode than for CHT95–Cu^2+^ solutions.

Next, two different needle sizes were used to investigate the influence on the size and morphology of CHT–copper (II) complex microspheres. It was expected that increasing the needle diameter would lead to the formation of larger particles [25]. Here, the smaller needle resulted in narrow size range of wet CHT85-Cu^2+^ microspheres, while samples obtained from CHT95 did not show any trend.

**Figure 4:**
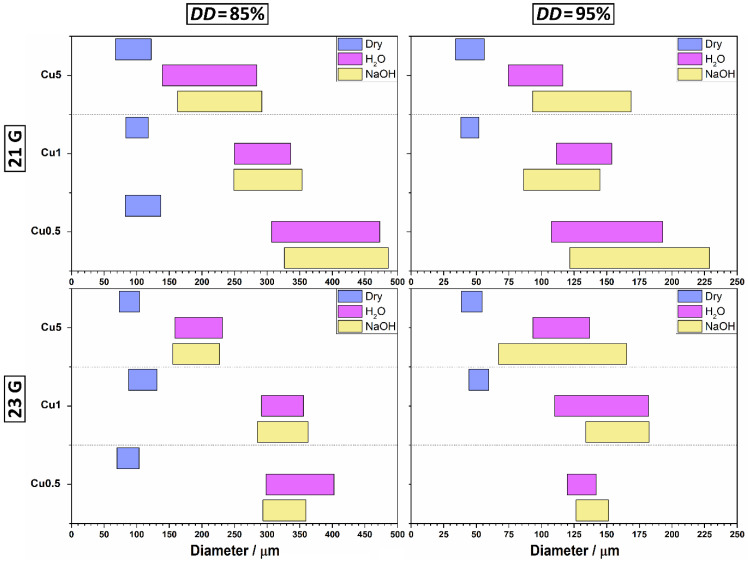
Estimated size of chitosan–Cu^2+^ microspheres in 5 wt.% NaOH solution, distilled water and dried microspheres.

The estimated diameters of prepared chitosan–Cu^2+^ microspheres are summarized in Figure 4. By increasing the concentration of cupric ions, the average diameters of wet CHT85-based microspheres decreased from 394.0 to 189.3 μm, and of CHT95-based microspheres from 165.8 to 111.6 μm (in NaOH solution). The average diameters of dry CHT85-based microspheres decreased slightly from 109.9 to 85.7 μm, and of CHT95-based from 53.2 to 42.8 μm. Furthermore, the size ranges of all prepared CHT-based microspheres became narrower with the increase of *c*(Cu^2+^). Furthermore, CHT85–Cu0.5 and CHT95–Cu0.5 complex microspheres obtained with 21 G needle had a wider size range in 5 wt.% NaOH solution and distilled water. It is interesting to note that the narrowest size range showed CHT85–Cu0.5 in alkali medium and CHT85–Cu1 in distilled water when 23 G needle was used. For CHT95-based microspheres, the narrowest size range in NaOH solution and distilled water had the complex with the lowest concentration of cupric ions (CHT95–Cu0.5). Dried CHT–Cu5 complexes microspheres prepared with 21 G needle showed the widest size range, for both used chitosans, while the narrowest had CHT85–Cu5 and CHT95–Cu1 microspheres obtained with 23 G and 21 G, respectively.

These changes could indicate the presence of stronger interaction between chitosan’s functional groups (–NH_2_ and –OH) and cupric ions with increasing *c*(Cu^2+^). Bai et al. [21] also observed smaller diameter of chitosan gel beads after immobilizing copper (II) ions onto beads, which was explained by the action of cupric ions as crosslinking agents in chitosan–Cu^2+^ beads. The formation of strong CHT–Cu^2+^ complex at higher *c*(Cu^2+^) could be responsible for the volume shrinking of microscale hydrogels.

Another interesting observation is the size of dried microspheres of all complexes. Even though the difference in size of wet microspheres was observed, dry microspheres showed sizes around 100 μm for CHT85-based complexes, and around 50 μm for CHT95-based complexes independent of copper (II) ions concentration. This result is very important when it comes to modulating the chemistry of microspheres by the metal concentration without affecting their sizes.

Overall, wet and dried CHT–Cu5 microspheres showed the most uniform size distribution and spherical shape, for both used chitosans.

### 3.4. Morphology of Dried Chitosan–Cu^2+^ Microspheres

The morphology of dried chitosan–Cu^2+^ microspheres was investigated by SEM.

As previously mentioned, CHT–Cu0.5 and CHT–Cu1 microspheres’ aggregates formed during the drying process were fragmented before SEM imaging, while CHT–Cu5 microspheres were imaged without previous processing. Furthermore, the morphology of CHT95–Cu0.5 complexes was not investigated.

**Figure 5:**
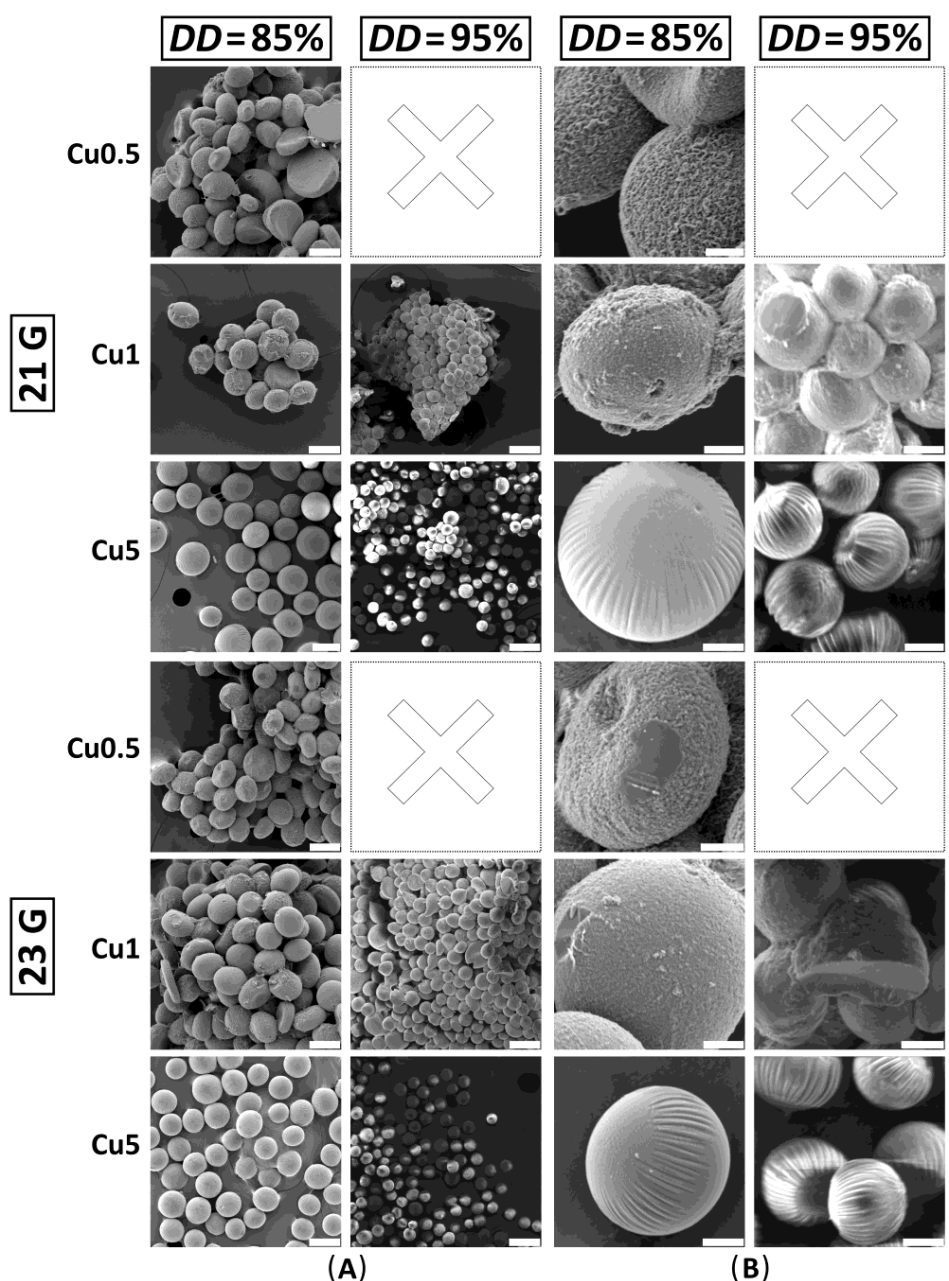
SEM micrographs of dried chitosan–Cu^2+^ microspheres. CHT95–Cu0.5 microspheres were not obtained after dehydration and drying process. (**A**) The scale bar is 100 μm. (**B**) The scale bar is 20 μm.

Figure 5 shows successfully obtained dried microspheres after dehydration with EtOH and drying with acetone.

SEM micrographs (Figure 5) show that all obtained samples are spherical and in agreement with microspheres’ sizes determined by the light microscope. Furthermore, changes in surface morphology of CHT-based microspheres with the addition of cupric ions can be observed.

The addition of Cu^2+^ ions at the lowest concentration (CHT85–Cu0.5) creates a rough, wrinkled surface. Furthermore, the concavity of some microspheres can be observed, which has been previously reported in the literature [20]. The same effect was also described as “hollow” particles [15] or “cavities” [1,10]. The formation of a wrinkled surface could be attributed to the addition of metal ions as reported by Liang et al. [38] where the surface roughness of chitosan microspheres was modulated by silver ions through self-assembly layers. The authors reported on micro- and nanoscale wrinkling structure on the microsphere surface as a result of different swelling behavior of chitosan and stiff Ag layer. Moreover, the thickness of Ag layer tuned the surface topographical patterns causing wrinkling of different sizes. Here, the addition of higher *c*(Cu^2+^) altered the topography of microspheres from rough to smoother onion-like surface for both chitosans.

Wet microspheres were dried by using EtOH and acetone. Such protocol was chosen to produce a non-porous interior of microspheres. As previously reported [7,33], when lyophilization was used as a drying step, the porous microstructure of irregularly shaped microspheres was obtained. The drying process highly affects the final microstructure and shape of microspheres, therefore, the usage of EtOH and acetone at ambient conditions in this work resulted in microspheres with good sphericity and uniform size.

### 3.5. Volume Ratio of Wet and Dry Microspheres

**Figure 6:**
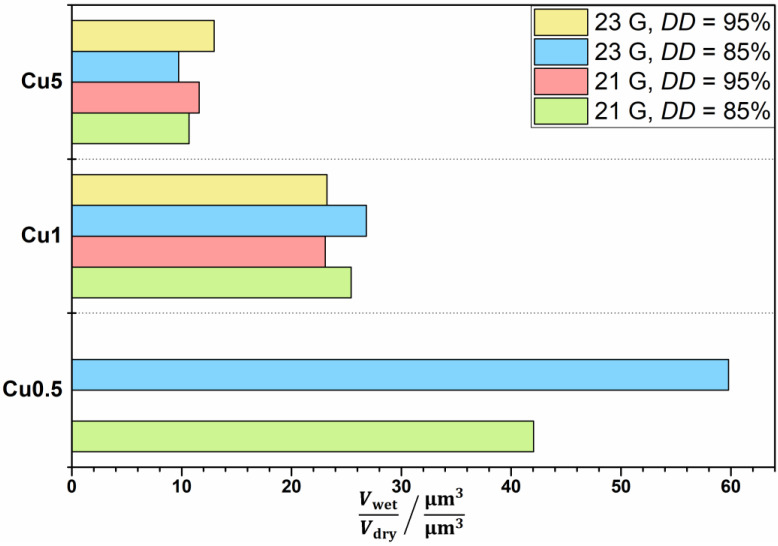
The estimated volume ratio (water content) of chitosan–Cu^2+^ complex microspheres.

The volume ratio was estimated as the ratio of wet microspheres in distilled water (*V*_wet_) and dried microspheres (*V*_dry_). Microspheres’ volume was estimated based on the mean value of at least 20 randomly chosen microsphere radii, assuming the total sphericity of the particles. As described previously, dry CHT95–Cu0.5 microspheres were not obtained after the drying process, hence the volume ratio was not assessed. Figure 6 shows the estimated volume ratio of obtained CHT–Cu^2+^ microspheres.

The volume ratio decreases at higher concentration of Cu^2+^ ions in CHT–Cu^2+^ complexes for both used chitosans. CHT85–Cu1 microspheres showed a slightly higher ability to absorb water with respect to CHT95–Cu1 samples, which is in accordance with the literature [5]. Ren et al. [5] concluded that swelling degree depends on deacetylation degree, i.e., that swelling capacity increases when *DD* decreases. Unlike CHT–Cu1 complexes, CHT–Cu5 complexes showed contrary results, i.e., slightly higher volume ratio was estimated for CHT95-based complexes.

The highest volume ratio showed microspheres with the lowest concentration of cupric ions (CHT85–Cu0.5), and the lowest had CHT–Cu5 complexes (for both used chitosans). This observation could be explained by the occupation of –NH_2_ and –OH groups by metal ions, i.e., by physical crosslinking of macromolecules through Cu^2+^ ions. Two types of interactions between chitosan and metal ion in chitosan–metal complex can be present: pendant and bridge model. If the bridge model is present, swelling is limited due to less possible active sites (less flexible network [39]) in chitosan for interactions with water molecules. Otherwise, if the pendant model is dominant, more amino groups are free and can make interactions with water molecules or –OH groups. According to Figure 6, microspheres with higher *c*(Cu^2+^) showed lower volume ratio which could be due to preferable interactions of chitosan functional groups with metal ions. The observed behavior is in agreement with viscosity results and estimated diameter.

### 3.6. Stability Study of Chitosan–Cu^2+^ Microspheres

**Figure 7:**
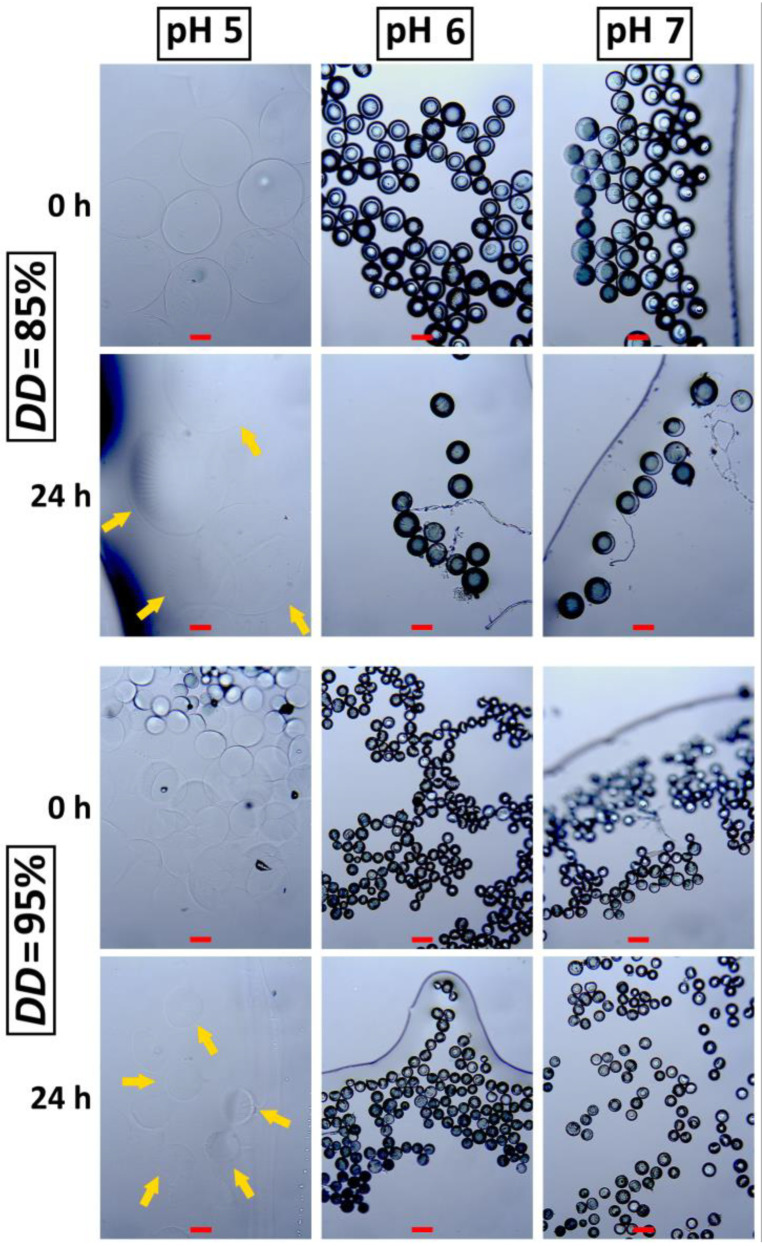
Micrographs of chitosan–Cu5 microspheres immersed in different buffer solutions at 0 and after 24 h. The scale bar is 100 μm.

Chitosan is soluble in the acidic environment, with pKa 6.2–7.0 [19]. The final pH of prepared chitosan solution in this work was 4.8. Based on that, the stability of prepared chitosan–Cu^2+^ was investigated in buffer solutions with pH values of 5, 6 and 7 after 24 h. CHT85–Cu5 and CHT95–Cu5 microspheres obtained by 23 G needle were chosen for stability evaluation due to narrow size range and high sphericity. The micrographs of chitosan–Cu^2+^ microspheres at the beginning (0 h) and after 24 h of immersion are shown in Figure 7.

CHT–Cu5 microspheres immediately swell and became transparent in buffer solution with pH 5 (0 h) indicating very high absorption capacity under slightly acidic conditions characteristic for chitosan-based materials. After 24 h of immersion, microspheres remained transparent and stable without significant changes in shape and size regarding the microspheres at the beginning of the immersion test. High and fast swelling in acidic solution is a result of amino groups’ protonation which usually ends in polymer dissolution. Our results indicate that greater amount of copper (II) ions could have occupied more amino groups that became unavailable for protonation and form stronger physical crosslink between chitosan chains. This is also evident at higher pH values (6 and 7) where immersed microspheres did not show any significant difference in size regarding the dried ones. Limited swelling capacity and good stability in acidic solution is characteristic for chemically crosslinked chitosan-based materials [28]; however, we have shown that physical interactions such as metal chelation could be a potential alternative to improve stability of chitosan microspheres.

## 4. Conclusions

The aim of this work was to produce stable chitosan-based microspheres through physical crosslinking via chelation interactions of copper with chitosan. Furthermore, the size, shape and morphology of chitosan–copper (CHT–Cu^2+^) complex microspheres obtained by the electrospraying process were investigated. The addition of cupric ions decreased the dynamic viscosity of pure chitosan solutions even at the lowest concentration of Cu^2+^ ions. The higher concentration of Cu^2+^ ions caused changes in the shape of microspheres, from slightly stretched droplets (CHT–Cu0.5 and CHT–Cu1) to round, smooth, more spherical CHT–Cu5 microspheres. Besides, higher *c*(Cu^2+^) was responsible for microspheres with smaller diameters. These changes could indicate the presence of stronger physical crosslinking between chitosan’s functional groups (–NH_2_ and –OH) and cupric ions which led to more homogeneous and spherically shaped microspheres. The morphology of microspheres has changed from a rough and wrinkled surface to a smoother, onion-like surface with the addition of Cu^2+^ ions. The wet/dry volume ratio decreased at higher concentration of Cu^2+^ ions, possibly due to preferable interactions of chitosan’s functional groups with copper (II) ions. Furthermore, the stability study indicated stable microspheres under slightly acidic conditions. It can be concluded that electrosprayed chitosan–copper (II) complex microspheres with non-porous interior show uniform size and good stability in aqueous medium.

## Figures and Tables

**Table 1:** Experimental conditions for electrospraying.

Parameter	Unit	Value
Concentration of acetic acid	%	0.5
Initial concentration of chitosan solution	wt.%	1.2
Flow rate of solution	mL h^−1^	5
Needle gauge	G	21 and 23
Applied voltage ^1^	kV	14 and 23
Dimensions of chamber (length/width/height)	cm	30/30/25
Distance between the needle tip and collector	cm	10
Concentration of NaOH	wt.%	5
Volume of NaOH (collector)	mL	50
Duration of electrospraying	min	60
Temperature (in the chamber)	°C	28 ± 1
Relative humidity (in the chamber)	%	65 ± 3

^1^ the applied voltage of 14 kV was used in electrospraying of CHT95-based complexes and voltage of 23 kV was set in the process for CHT85-based complexes.

**Table 2:** FTIR bands characteristic for chitosan and chitosan–Cu^2+^ microspheres.

**Sample**	**Absorption Band/cm^−1^**									
	–OH	–NH_2_	C–H		Amide I	Amide II	Amide III	C–O–C	–COH	
CHT85	3362	3290	2916	2872	1650	1541	1336	1151	1066	1030
CHT85–Cu0.5	3358	3289	2914	2873	1650	1542	*	1150	1061	1025
CHT85–Cu5	3354	3282	2924	2873	1648	1542	1337	1151	1056	1020
CHT95	3359	3292	2919	2872	1650	1541	1336	1151	1065	1029
CHT95–Cu0.5	3363	3290	2921	2874	1651	1541	1339	1150	1061	1027
CHT95–Cu5	3361	3275	2920	2873	1649	1541	1339	1151	1056	1021

* unable to detect due to band overlapping.

**Table 3:** Dynamic viscosity of pure chitosan solutions (CHT85 and CHT95) and CHT–Cu^2+^ complex solutions.

Sample	*η*/mPa s	Sample	*η*/mPa s
CHT85	90.1 ± 0.1	CHT95	24.1 ± 0.1
CHT85–Cu0.5	59.5 ± 0.2	CHT95–Cu0.5	17.8 ± 0.1
CHT85–Cu1	59.6 ± 0.4	CHT95–Cu1	16.7 ± 0.1
CHT85–Cu5	38.6 ± 0.1	CHT95–Cu5	15.0 ± 0.1

## Data Availability

Not applicable.
